# Artificial Intelligence Applications in Mental Health: A Systematic Review of Clinical Practice, Educational Transformation, and Ethical Governance

**DOI:** 10.3390/healthcare14172721

**Published:** 2026-08-26

**Authors:** Rania Maher Alhalawany, Yahya Mubarak Khatatbeh, Aeshah Ali Jawkhab

**Affiliations:** 1 Department of Health Sciences, College of Health and Rehabilitation Sciences, Princess Nourah Bint Abdulrahman University, P.O. Box 84428, Riyadh 11671, Saudi Arabia; rmalhalawany@pnu.edu.sa (R.M.A.); aajookhap@pnu.edu.sa (A.A.J.); 2 Department of Psychology, College of Social Science, Imam Mohammad Ibn Saud Islamic University (IMSIU), P.O. Box 5701, Riyadh 11432, Saudi Arabia

**Keywords:** artificial intelligence, mental health, psychiatry, psychology, machine learning, chatbots, digital mental health, ethical governance, systematic review, generative AI

## Abstract

**Background**: Artificial intelligence (AI) is one of the most influential technological innovations in contemporary mental healthcare. Advances in machine learning, natural language processing, conversational agents, and large language models have accelerated the integration of AI into clinical practice, professional education, and healthcare. Despite its increasing adoption, important questions remain regarding its clinical effectiveness, implementation, safety, and ethical governance. **Objective**: This systematic review aimed to synthesize the current evidence on the application of artificial intelligence in mental health, with particular emphasis on clinical practice, educational transformation, and ethical governance. **Methods**: This systematic review was conducted in accordance with the PRISMA 2020 guidelines. PubMed/MEDLINE, Scopus, Web of Science, PsycINFO, and Google Scholar were systematically searched. The electronic database search was last conducted on 31 December 2025, and studies published between January 2019 and December 2025 were considered eligible. Eligible studies examined the application of artificial intelligence in mental health across clinical practice, educational contexts, and ethical governance. Study selection, data extraction, and methodological quality assessment were carried out independently by two reviewers using predefined eligibility criteria and standardized extraction forms. Owing to substantial methodological heterogeneity across the included studies, the findings were synthesized narratively. **Results**: A total of 88 studies met the eligibility criteria and were included in the final qualitative synthesis. The findings showed that AI demonstrated potential to improve diagnostic support, risk prediction, treatment planning, symptom monitoring, and access to psychological support. AI also supported educational innovation and workforce development while highlighting the importance of ethical governance for responsible implementation in mental healthcare. **Conclusions**: Future progress will depend on interdisciplinary collaboration to ensure that AI complements rather than replaces human expertise. Although AI demonstrates substantial potential, many systems remain experimental, with limited external validation. Prospective multicenter evaluation, transparent algorithm development, and robust ethical governance are therefore essential before widespread clinical implementation.

## 1. Introduction

Mental health disorders represent one of the most significant public health challenges worldwide, affecting people of all ages and socioeconomic groups. The growing global burden of mental disorders has intensified the demand for scalable, accessible, and evidence-based mental healthcare solutions. The World Health Organization estimates that prior to the COVID-19 pandemic, around one billion people suffered from a mental disorder, and more recent estimates suggest that anxiety and depressive disorders increased substantially during and following the pandemic. Mental health conditions extend beyond individual health and account for a large part of global disability, diminished quality of life, social exclusion, economic burden and premature mortality. Furthermore, suicide remains a major public health concern, accounting for more than 700,000 deaths annually and ranking among the leading causes of death for young adults globally [1].

Despite advances in psychiatric care and psychological interventions, substantial treatment gaps persist in many health systems, particularly in low- and middle-income countries, where shortages of mental health professionals, limited resources, persistent stigma, and inequitable access continue to restrict timely and effective care [2].

As the global burden of mental illness continues to increase, there is a growing need for innovative strategies to improve prevention, diagnosis, treatment, and long-term management. Against this background, artificial intelligence (AI) has emerged as one of the most influential technological innovations in modern healthcare. Artificial intelligence encompasses machine learning, deep learning, natural language processing, predictive analytics, and generative AI. In practice, these tools enable the analysis of large and complex datasets, helping to reveal patterns and relationships that may be difficult to detect using conventional statistical methods or standard clinical pathways [3,4,5].

The evolution of artificial intelligence in mental health care has moved from rule-based decision support systems to sophisticated machine learning algorithms and deep learning architectures and, more recently, to the emergence of generative AI and large language models. This technological evolution has expanded the potential role of AI from automated data processing to predictive analytics, clinical decision support, personalized treatment planning, digital phenotyping, conversational agents and adaptive educational platforms. However, the increasing complexity of the algorithms has also raised important scientific and implementation challenges, including model interpretability, external validation, fairness across diverse populations, and regulatory oversight, all of which should be carefully considered before widespread clinical deployment [4,6,7,8].

Machine learning algorithms can complement conventional clinical approaches by detecting subtle, nonlinear relationships within multidimensional datasets [9,10,11]. In parallel, these developments have contributed to the rise of precision psychiatry, which aims to tailor prevention and treatment strategies to individual patient characteristics rather than applying generalized approaches [9,10,12].

Advances in natural language processing and large language models have accelerated the development of conversational agents and mental health chatbots capable of providing psychoeducation, emotional support, and self-management assistance [13]. Although these technologies may improve accessibility to mental health services, concerns remain regarding safety, reliability, and clinical appropriateness in high-risk situations [11,14]. AI has demonstrated considerable potential to transform multiple aspects of mental healthcare delivery, in addition to the improvement of diagnostic support and service access. Machine learning algorithms have shown promise in identifying complex interactions between biological, psychological, behavioral and environmental factors contributing to psychiatric disorders, thus supporting earlier detection of risks and more personalized planning of treatment. AI-supported clinical decision support systems may also facilitate evidence-based selection of treatment, optimize resource allocation and improve continuity of care through ongoing patient monitoring. However, current evidence is heterogeneous, with a high degree of variation in study design, datasets, validation procedures and performance measures reported, limiting generalizability of many published results [4,6,10,15]. This heterogeneity also suggests that apparently similar AI applications may yield different mental health outcomes across populations and clinical settings, underscoring the importance of comparing both convergent and divergent findings rather than relying solely on reported technical performance.

Adaptive learning platforms, intelligent tutoring systems, virtual patient simulations, and AI-assisted training settings have all been proposed as ways to improve clinical education and prepare the workforce [11,16,17,18]. Despite these potential benefits, implementing AI in mental health remains complex and raises substantial ethical, legal, and governance challenges. The collection and analysis of sensitive mental health data raise concerns regarding privacy, confidentiality, informed consent, and data security. At the same time, AI systems may inherit and then amplify biases embedded in training datasets, potentially producing inequitable outcomes across demographic groups. There is also ongoing uncertainty about transparency, explainability, accountability, regulatory supervision, and even the public’s trust in AI-supported decisions [7,8,19]. As AI becomes increasingly integrated into clinical practice, education, and health system management, robust governance and regulatory frameworks are needed so that implementation remains safe, fair, and genuinely responsible.

Despite these advances, many important scientific and translational challenges remain to be addressed. Several published AI models have been trained on relatively small or highly selective datasets and have limited external validation, raising concerns about their robustness across different clinical settings and populations. Differences in methodological quality, reporting standards, outcome measures and evaluation frameworks make comparisons between studies difficult and impede translation into routine clinical practice. These limitations highlight the need for a comprehensive synthesis of evidence that can critically integrate clinical, educational and governance perspectives in a single analytical framework [3,7,8,10,15].

Even though the literature on AI in mental health has expanded rapidly, the overall evidence remains fragmented across multiple areas. Earlier reviews often focused on specific areas, such as machine learning models, digital interventions, chatbot applications, or ethical issues, which are frequently handled in isolation [3,15,20].

As a result, it remains unclear how AI in general affects clinical practice, educational transformation, and governance structures that support mental health systems. This fragmentation makes it difficult for clinicians, educators, researchers, and policymakers to develop an integrated understanding of AI adoption across the broader mental healthcare landscape. Moreover, limited attention has been given to systematically comparing areas of convergence and divergence across mental health findings, particularly in relation to differences in study populations, AI methodologies, validation strategies, and clinical contexts. Furthermore, this review sought to compare areas of convergence and divergence across the included evidence, identify current evidence gaps and emerging challenges, and define future research priorities to support the responsible implementation of AI in mental healthcare [3,15,20].

However, limited evidence synthesis has simultaneously examined clinical applications, educational transformation, and governance frameworks within a single integrated review, particularly in the context of recent advances in generative AI and large language models [4,6]. To ensure analytical clarity while retaining the multidisciplinary scope of the review, the synthesis was mapped onto three pre-specified analytical domains prior to study selection and data extraction: (1) clinical applications of artificial intelligence in mental healthcare, (2) educational transformation and professional training, and (3) ethical, legal and governance issues. This analytical structure was adopted to enable a focused synthesis within each of the domains, while retaining an integrated perspective on the role of artificial intelligence across existing mental health systems.

Although previous reviews have examined individual aspects of AI in mental health, such as machine learning, chatbots, digital interventions, or ethical issues, no recent systematic review has comprehensively synthesized evidence across clinical practice, educational transformation, and ethical governance while incorporating recent advances in generative AI and large language models. This review was designed to address this gap.

## 2. Materials and Methods

### 2.1. Study Design

This study was conducted as a systematic review to critically examine contemporary applications of artificial intelligence (AI) in mental health, with a focus on clinical practice, educational transformation, and ethical governance. This review was conducted according to the Preferred Reporting Items for Systematic Reviews and Meta-Analyses [21] (PRISMA 2020) standards to guarantee methodological rigor, transparency, and reproducibility throughout the review process. Given the rapid development of AI technologies and their increasing use in mental healthcare systems, a systematic review method was deemed suitable to synthesize the current body of evidence and identify emerging trends, opportunities, and challenges across these domains.

The distinctive contribution of the present systematic review lies in its multidisciplinary analytical framework. Previous systematic reviews have primarily focused on isolated applications of artificial intelligence, such as machine learning algorithms, conversational agents, digital interventions, or ethical issues separately. In contrast, the present review was designed to provide an integrated synthesis across three complementary domains: clinical practice, educational transformation, and ethical governance. The analytical framework was established a priori and informed the literature search, study selection, data extraction, and narrative synthesis. By synthesizing evidence across these interconnected domains, the review provides a more comprehensive understanding of the implementation of artificial intelligence in contemporary mental healthcare and identifies evidence gaps that may not be apparent when each domain is examined independently [3,15,20]. The review design was structured to permit systematic comparison of both convergent and divergent findings across heterogeneous clinical, educational, and governance evidence while preserving the methodological distinctions among the included study designs.

#### Protocol and Registration

This systematic review was conducted in accordance with the Preferred Reporting Items for Systematic Reviews and Meta-Analyses guidelines (PRISMA 2020). Prior to conducting the review, the study objectives, eligibility criteria, search strategy, screening procedures, data extraction framework, and quality assessment approach were predefined to ensure methodological consistency and transparency throughout the review process. However, the review protocol was not prospectively registered in PROSPERO or any other public registry. This decision should be considered when interpreting the methodological transparency of the review.

### 2.2. Search Strategy

A comprehensive systematic search was conducted to identify studies examining the application of artificial intelligence (AI) in mental health clinical practice, mental health education, and ethical governance. The electronic database search was last conducted on 31 December 2025. Studies published between January 2019 and December 2025 were considered eligible for inclusion.

To ensure methodological reproducibility, database-specific search strategies were developed for each electronic database. Although the conceptual search framework remained consistent across all databases, the search syntax, field tags, controlled vocabulary, truncation symbols, and indexing terms were adapted according to the requirements of Scopus, Web of Science Core Collection, PubMed/MEDLINE, PsycINFO, and Google Scholar. The complete database-specific search strategies are provided in Table 1.

To maximize retrieval of relevant studies, both controlled vocabulary, where applicable, and free-text keywords were used. Search terms were combined with mental health-related terms such as “mental health,” “psychiatry,” “psychology,” “depression,” “anxiety,” and “psychotherapy” with artificial intelligence-related terms such as “artificial intelligence,” “machine learning,” “deep learning,” “natural language processing,” “large language models,” “generative AI,” “ChatGPT,” and “mental health chatbots.” Additional terms related to education, training, ethics and governance were also included. Boolean operators were used to combine search terms, and the search strategy was adapted to the indexing requirements of each database.

Where supported by the database interface, search filters were applied to restrict retrieval to peer-reviewed publications, articles published in English, human studies where applicable, and the predefined publication period (January 2019–December 2025). No restrictions were applied regarding country of origin or study design during the initial search.

The generic search strategy applied across all databases is presented below, whereas the complete database-specific electronic search strategies are provided in Supplementary Material S1.

(“artificial intelligence” OR AI OR “machine learning” OR “deep learning” OR “natural language processing” OR “large language model*” OR “generative AI” OR ChatGPT OR chatbot*) AND (“mental health” OR psychiatry OR psycholog* OR depression OR anxiety OR psychotherapy).

The same conceptual search strategy was used in Google Scholar. For consistency, reproducibility, and feasibility, screening was limited to the first 200 records ranked by relevance, consistent with common practice in systematic reviews using Google Scholar. During the title and abstract screening stage, duplicate records and obviously irrelevant citations were excluded.

Grey literature, such as dissertations, conference abstracts, preprints, technical reports, policy documents, and other non-peer-reviewed sources, was not included due to the review’s decision to focus solely on peer-reviewed scientific publications in order to ensure methodological consistency and evidence quality. This decision was predefined in the review protocol and applied consistently throughout the study selection process.

### 2.3. Eligibility Criteria

Searches were carried out to identify studies published in the English language between January 2019 and December 2025 that explored the use of artificial intelligence in mental health settings. A wide variety of evidence sources was deliberately included in order to provide a comprehensive overview of the field, reflecting the interdisciplinary and rapidly evolving nature of artificial intelligence research. Eligible publications encompassed primary empirical studies (randomized controlled trials, observational studies, cross-sectional studies, mixed-methods studies and technology development studies) and secondary evidence (systematic reviews, meta-analyses, scoping reviews and high-quality narrative reviews). Primary studies were included for the evaluation of original empirical evidence; review-level publications were included to identify overarching research trends, summarize existing evidence and contextualize emerging developments. Primary and secondary evidence were identified and interpreted separately during data extraction and narrative synthesis in order to reduce evidence duplication and improve clarity of interpretation.

Studies were required to address at least one of the following domains: (1) clinical applications of artificial intelligence in mental health; (2) educational transformation and professional training; or (3) ethical, legal, and governance aspects related to the implementation of AI in mental health settings. Studies focusing exclusively on technical algorithm development without a direct application to mental health were excluded.

### 2.4. Study Selection Process

Two reviewers independently screened the titles and abstracts against the predefined eligibility criteria. Potentially eligible studies were then assessed through full-text review. Disagreements between reviewers were resolved through discussion and consensus, with consultation from a third reviewer when necessary. The study selection process was conducted in accordance with the PRISMA 2020 guidelines. To enhance transparency and reproducibility, the complete database-specific electronic search strategies are provided in Supplementary Material S1, and study selection procedures were implemented in accordance with the PRISMA 2020 recommendations. Reasons for exclusion were documented at each stage of screening and eligibility assessment. The final study selection results are reported in Section 3 and summarized in the PRISMA flow diagram.

### 2.5. Data Extraction

Data extraction was performed independently by two reviewers using a standardized data extraction form to ensure consistency and accuracy. Any discrepancies were resolved through discussion and consensus with consultation from a third reviewer when necessary. The relevant data extracted from each study included author information, year of publication, country, study design, sample characteristics, artificial intelligence technology used, mental health domain, major outcomes, main results, and limitations stated. Special emphasis was placed on the specification of the individual AI techniques used, such as machine learning, deep learning, natural language processing, large language models, conversational agents, and generative AI systems. Data on therapeutic efficacy, educational applications, ethical issues, and governance implications were documented for thematic synthesis. To facilitate comparison across studies, both qualitative and quantitative study characteristics were extracted and compared during data extraction. This comparative extraction approach was used to identify similarities and differences in reported mental health outcomes, methodological characteristics, validation status, and implementation-related limitations across studies.

Particular attention was given to reported improvements of AI over conventional approaches, including diagnostic accuracy, early risk detection, treatment planning, accessibility to mental health services, symptom monitoring, and clinical decision support. Where available, quantitative performance measures (e.g., accuracy, sensitivity, specificity, area under the receiver operating characteristic curve [AUC], and reported effect estimates) were extracted to support study-level comparison. The extracted information was reviewed for consistency and completeness prior to thematic synthesis and analysis.

Classification of studies was not based on mutually exclusive categories. Individual studies frequently addressed multiple mental health conditions, applied more than one artificial intelligence technique, or contributed to more than one thematic domain. For descriptive purposes in Table 2, each study was classified according to its primary objective, dominant artificial intelligence methodology, and principal mental health application as identified by the study authors. Secondary characteristics were retained during data extraction and were considered throughout the narrative synthesis, although they were not simultaneously displayed within the summary table to maintain clarity and avoid duplication.

Data extraction was pilot-tested on a sample of eligible studies before full extraction to improve consistency between reviewers.

### 2.6. Methodological Quality Assessment

Methodological quality was independently assessed by two reviewers using validated critical appraisal tools appropriate for each study design. Randomized controlled trials were assessed using the Cochrane Risk of Bias 2 (RoB 2) tool, whereas non-randomized intervention studies were evaluated using ROBINS-I. Observational and cross-sectional studies were appraised using the Joanna Briggs Institute (JBI) Critical Appraisal Checklists. Mixed-methods studies were assessed using the Mixed Methods Appraisal Tool (MMAT, 2018 version). Systematic reviews and meta-analyses were evaluated using the AMSTAR 2 instrument, while narrative reviews were appraised using the Scale for the Assessment of Narrative Review Articles (SANRA). Technology development studies were assessed using the most appropriate JBI critical appraisal checklist according to their underlying study design. Each study was independently evaluated by two reviewers, and any disagreements were resolved through discussion and consensus, with consultation from a third reviewer when necessary. Quality assessment findings informed the interpretation of the evidence but were not used as exclusion criteria. A detailed summary of the methodological quality assessment for each included study is provided in Supplementary Material S2.

### 2.7. Data Synthesis

Because of the substantial methodological heterogeneity across the included studies, a quantitative meta-analysis was neither appropriate nor feasible. Primary empirical studies and review-level evidence were synthesized separately throughout the narrative synthesis to minimize evidence duplication and to facilitate a clearer interpretation of the available literature. Therefore, a structured narrative synthesis was undertaken. To improve analytical clarity and reduce conceptual heterogeneity, the synthesis was guided by a predefined analytical framework established before data extraction. The evidence was thematically mapped onto three independent domains, congruent with the review objectives, namely (1) clinical applications of artificial intelligence in mental healthcare, (2) educational transformation and professional training, and (3) ethical, legal and governance issues. Each domain was independently synthesized and critically interpreted, and then the findings were integrated to generate an overall understanding of artificial intelligence implementation in mental health. The domain-based analytical framework enabled a more focused assessment of evidence within each area and also retained the multidisciplinary scope of the review. Methodological quality was taken into account throughout the interpretation of findings within each domain.

Within each domain, studies were systematically compared according to study design, participant characteristics, artificial intelligence methodology, reported outcomes, performance measures, and implementation challenges to identify areas of agreement, divergence, and remaining evidence gaps.

Comparative interpretation focused on whether findings were directionally consistent across studies and on identifying methodological or contextual factors that could explain divergent findings. Particular consideration was given to differences in study design, population characteristics, AI techniques, outcome definitions, validation procedures, and clinical settings. Given the heterogeneity of the evidence, these comparisons were interpreted narratively rather than as pooled quantitative estimates.

### 2.8. Ethical Considerations

Ethical approval was not required because this study was based exclusively on the analysis and synthesis of previously published literature and did not involve human participants, identifiable personal data, or clinical interventions. The review was conducted in accordance with the established principles of responsible research and systematic review methodology. Particular attention was given to synthesizing ethical issues discussed in the included studies, including privacy, transparency, accountability, algorithmic bias, explainability, and responsible governance of artificial intelligence in mental health.

## 3. Results

### 3.1. Study Selection Results

**Figure 1:**
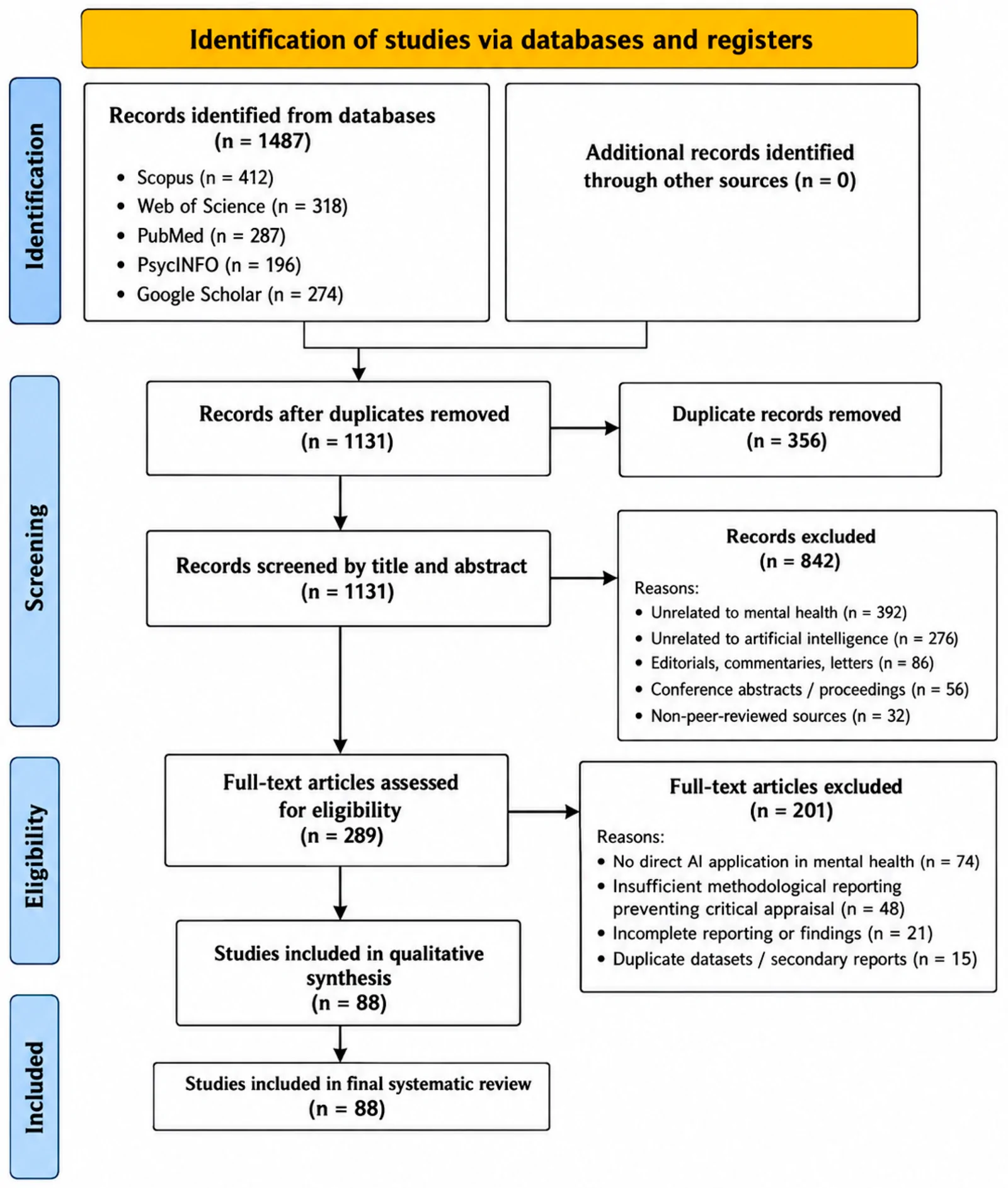
PRISMA 2020 flow diagram of the study selection process.

A total of 1487 records were identified through database searching in Scopus, Web of Science, PubMed/MEDLINE, PsycINFO, and Google Scholar. After removing 356 duplicate records, 1131 unique records remained for title and abstract screening. During screening, 842 records were excluded because they did not meet the predefined eligibility criteria. The full texts of 289 articles were assessed for eligibility. Of these, 201 articles were excluded because they did not address the predefined review domains, exhibited insufficient methodological reporting preventing critical appraisal, provided inadequate outcome reporting, or did not meet the other predefined eligibility criteria. Ultimately, 88 studies met the eligibility criteria and were retained for the final qualitative synthesis. The study selection process is summarized in Figure 1.

### 3.2. Quality Assessment

The methodological quality of all 88 included studies was evaluated using validated critical appraisal tools for each study design as presented in the Methods. The overall methodological quality of the included evidence was moderate to high. Most studies showed acceptable methodological rigor, including well-defined objectives, appropriate study design, transparent reporting and conclusions supported by the reported findings. Differences in methodological quality were noted across different study designs, especially in relation to sample characteristics, completeness of reporting, and validation of artificial intelligence models. Quality assessment findings were considered throughout the narrative synthesis to ensure that the interpretation of evidence reflected the methodological strengths and limitations of the included studies. No study was excluded solely on the basis of its quality assessment after meeting the predefined eligibility criteria. A detailed quality appraisal for each included study is presented in Supplementary Material S2.

### 3.3. Characteristics of Included Studies

A total of 88 studies met the predefined eligibility criteria and were included in the final qualitative synthesis. The included studies were published between 2019 and 2025, reflecting the rapid expansion of research on artificial intelligence in mental health. After 2022, publication activity increased considerably, and more than half of the included studies were published between 2023 and 2024.

Regarding the origin of the studies, the geographical distribution showed a clear dominance of high-income countries. The United States, the United Kingdom, Canada, Australia, and several European countries together made up most of the publications. However, an expanding evidence base from Asia and the Middle East was also observed. Taken together, these findings imply that the development and evaluation of AI-enabled mental health technologies are strongly shaped by regions that already have advanced digital infrastructure, relatively high research funding, and established healthcare innovation ecosystems.

Methodologically, the included studies were diverse. They included systematic reviews, randomized controlled trials, observational studies, cross-sectional work, qualitative research, mixed-methods designs, and technology development investigations. This diversity reflects the multidisciplinary nature of artificial intelligence research in mental health and the range of approaches used to examine effectiveness, usability, implementation, ethical issues, and educational outcomes. Machine learning was the most frequently explored AI approach, followed by deep learning, natural language processing, conversational agents, and large language models. These systems have been used for a broad set of mental health conditions, such as depression, anxiety disorders, suicidal behavior, schizophrenia, bipolar disorder, stress-related issues, and general psychological well-being.

In addition, chatbots and conversational agents are among the most heavily studied applications because they are scalable, relatively easy to access, and may support continuous mental health care. First, AI technologies are increasingly being used in clinical practice for diagnosis, prediction, support for treatment, and patient monitoring. Second, AI has helped reshape education through simulation-based learning, adaptive educational systems, and professional training in psychology and psychiatry. Third, ethical governance has become a major area of focus. Many studies have underscored privacy safeguards, algorithmic bias, transparency, accountability, and regulatory oversight as necessary building blocks for responsible AI rollouts in mental health contexts. The included evidence comprised both primary empirical studies and evidence-synthesis publications, reflecting the multidisciplinary and rapidly evolving nature of artificial intelligence research in mental health. To maintain analytical consistency, the findings are presented according to the three predefined domains established during the review process. Clinical, educational, and governance-related evidence were synthesized and interpreted separately before being integrated into the overall narrative, thereby facilitating a more structured and focused interpretation of the heterogeneous literature.

Across these domains, the included studies showed both convergent and divergent patterns, with the degree of consistency varying according to study design, AI methodology, population characteristics, outcome measures, and validation approach.

As shown in Table 2, the included studies were published between 2019 and 2025, reflecting sustained growth in scholarly interest in artificial intelligence applications within mental health. Machine learning and deep learning represent the most frequently investigated technologies, whereas depression, anxiety, and suicide risk assessment remain among the most commonly studied application domains.

### 3.4. AI Applications in Clinical Practice

AI is transforming mental health care through its applications in diagnosis, risk prediction, clinical decision support, therapy delivery, symptom monitoring, and patient engagement. The studies reviewed here suggest that AI systems have the potential to enhance the accessibility, efficiency, and personalization of mental health services, and to overcome workforce shortages and the increasing demand for psychological and psychiatric care.

The early detection and diagnosis of mental health conditions using machine learning and deep learning algorithms is one of the most investigated clinical applications. The reviewed evidence shows that AI models can detect depression, anxiety, bipolar disorder, schizophrenia, and suicidal behavior based on electronic health records, neuroimaging data, behavioral measures, speech features, and digital communication data [22]. The studies ranged from exploratory observational investigations to externally validated prediction models. Machine learning algorithms were usually evaluated with retrospective clinical data sets, and few studies reported external validation or prospective clinical assessment. Diagnostic performance was most commonly evaluated by accuracy, sensitivity, specificity and the area under the receiver operating characteristic curve (AUC), with substantial variation across the study populations and AI models.

Several AI-based prediction models demonstrated promising performance in detecting clinically relevant patterns that may be difficult to identify using conventional assessment approaches. These results indicate the potential of AI technologies as useful decision-support tools for identifying at-risk individuals and providing timely intervention.

Another important application is in predictive analytics and risk assessment. Several studies have applied machine learning for the prediction of mental health outcomes, such as suicide attempts, worsening of symptoms, relapse, hospitalization, and treatment non-adherence. AI systems were able to learn complex relationships of biological, psychological, and social risk factors through the integration of vast and heterogeneous information. Predictive capabilities may help clinicians prioritize high-risk patients, target interventions appropriately, and improve resource allocation in preventive psychiatry and individualized mental healthcare. However, model generalizability, external validation, and algorithmic fairness are major issues that require further study.

The reviewed evidence included observational studies, retrospective cohort analyses, and a limited number of clinically validated prediction models. Although predictive performance was generally encouraging, only a small proportion of studies evaluated model performance in independent external populations, highlighting the need for broader clinical validation before routine implementation.

The analysis also revealed a significant increase in mental health assistance through conversational agents, virtual therapists, and AI-powered chatbots. These systems increasingly use natural language processing and large language models, engage users in interactive discussions, and provide psychoeducation, cognitive behavioral therapy-based therapies, and emotional support. Research suggests that chatbot-based therapies can help reduce anxiety, stress, and mild depressive disorder symptoms and improve access to care for populations facing geographical, financial, or social barriers. Their 24/7 availability and timely responses are also important advantages. The majority of the included studies suggested that chatbots should be used to support qualified mental health practitioners, especially in the case of crises or serious psychiatric illnesses.

However, most published evidence was derived from pilot studies, feasibility studies, or short-term clinical evaluations, with relatively few randomized controlled trials demonstrating sustained clinical effectiveness. Consequently, current evidence primarily supports the adjunctive use of AI-powered chatbots rather than their independent implementation in routine psychiatric care.

Another common clinical use is computerized phenotyping and continuous monitoring. Mobile health technologies, wearable devices, and sensor-based systems can potentially collect real-time behavioral and physiological data for analysis using AI. The studies included in this review showed the potential use of these technologies for monitoring mood, sleep, social interaction, physical exercise, and emotional states. The majority of studies assessing digital phenotyping were observational or proof-of-concept studies, with a limited number of studies reporting longitudinal and/or externally validated clinical findings. Current evidence should be viewed as preliminary, and further prospective validation is required before these technologies can be implemented into routine clinical mental health practice. The use of multiple data sources may enable more dynamic and personalized assessment of mental health trajectories and support early intervention before clinical deterioration occurs. However, data protection, informed consent, and long-term user acceptance should be carefully considered during implementation.

Findings indicate that AI tools hold promise for bolstering clinical mental health care, mainly via better diagnostic accuracy, forecasting analytics, improved access to therapy, and patient monitoring. However, the reviewed literature emphasizes that deploying AI successfully depends on strict clinical validation, open and transparent algorithm design, careful ethical safeguards, and continuous human supervision. Therefore, AI should be viewed as an adjunct to clinical expertise rather than a replacement for professional judgment or therapeutic relationships. Nevertheless, the current evidence indicates that many AI systems remain at an early stage of clinical translation and require rigorous prospective validation before routine implementation across diverse clinical settings.

The clinical findings demonstrated both convergent and divergent patterns across the included evidence. Evidence from multiple sources supported the potential for AI to enhance diagnostic support, risk prediction, symptom monitoring, treatment decision support, and access to mental health services. By contrast, we observed variability in the size and consistency of benefits reported, especially across studies with different population characteristics, study design, AI methodology, and validation procedures. While some studies have shown promising predictive or diagnostic performance, others have shown limited generalizability, algorithmic bias, false positive risks, and insufficient prospective or externally validated evidence. Hence, while the evidence supports the potential utility for AI-assisted clinical applications, the reproducibility and clinical translation of these benefits depend on the quality of the methodology and external validation.

As shown in Table 3, the representative clinical evidence demonstrates a consistent pattern of promising AI-supported benefits accompanied by substantial variation in validation, generalizability, safety, and methodological robustness across applications.

Table 3 presents representative studies linking the synthesized clinical applications to the underlying evidence, including psychiatric diagnosis, suicide-risk prediction, treatment-response prediction, conversational agents, and symptom monitoring. Additional study-level characteristics of the included evidence are provided in Appendix A (Table A1).

### 3.5. AI in Educational Transformation and Professional Training

Artificial intelligence in education has transformed mental health practitioner training and development. The literature review identified AI technologies as important tools to improve educational efficiency, personalized learning, clinical competence development, and access to specialist mental health training. These improvements are important, given the increasing demand for mental health care and the need to educate highly competent staff who can navigate complex clinical contexts.

The most popular educational application in the fields of psychology and psychiatry was AI-assisted learning. It was demonstrated that adaptive learning systems based on machine learning algorithms can adapt instructional materials to the performance, knowledge gaps, and preferences of students. Personalized feedback, adaptive assessment, and customized learning paths increased students’ engagement and retention. The reviewed research shows that AI-assisted educational settings can help students and trainees acquire theoretical information more efficiently and address their different learning demands.

Simulation-based schooling is another innovation. Artificial intelligence has been used to develop virtual patients, intelligent tutoring systems, and immersive training environments simulating clinical settings. These technologies enable psychology and psychiatry trainees to conduct diagnostic interviews, risk assessments, clinical decision-making, and therapeutic communication in safe and controlled environments. Several studies have shown across the reviewed evidence that AI-driven simulation technologies improve learner confidence, clinical reasoning, and practical skills while reducing the need for resource-intensive traditional training.

The reviewed literature indicates a growing role for conversational AI and virtual mentors in professional training. AI-based conversational platforms have been used to support self-directed learning, improve access to educational resources, and provide timely responses to clinical and theoretical questions. These tools were seen as beneficial in continuing professional development programs because they can provide flexible, on-demand learning help to healthcare workers. Furthermore, the emergence of large language models made it possible to create more nuanced teaching exchanges and better information retrieval. AI has also been applied in educational administration and curriculum design and even in supporting students. A number of studies have leaned on predictive analytics to identify children who appear at risk for academic difficulties, estimate learning outcomes, and adapt instructional approaches as needed.

In these contexts, institutions can design curricula, manage resource allocation, and support learners using data-informed approaches. Therefore, AI may improve outcomes in mental health education and overall system performance. However, in the reviewed studies, several challenges were identified when bringing AI into mental health education. Studies highlighted concerns regarding overdependence on automated systems, possible weaknesses in AI-generated educational material, fewer opportunities for interpersonal learning, and the need to keep critical thinking abilities strong in trainees. In addition, ethical concerns have arisen regarding data privacy, transparency, algorithmic bias, and educational equity.

Taken together, these points show why it is important to treat this AI technology like educational tools, not like a replacement for professional oversight, human guidance, or experiential learning in practice. Our results indicated that AI is transforming mental health education through the provision of personalized education and training, improved simulation-based education and training, and professional development support. Successful implementation in education needs careful governance, continuous evaluation, and the use of responsible implementation strategies that preserve the critical role of human educators and capitalize on the unique benefits of AI technology.

The studies reviewed were generally in agreement about the promise of AI to support personalized learning, simulation-based training and professional development in mental health education. However, there are still important questions that remain unanswered about the long-term educational impact of AI-supported learning, how competencies learned in simulation are transferred to real clinical practice, and the impact of AI-supported learning on critical thinking and professional judgement. Much of the current evidence comes from education evaluations, pilot implementations and short-term studies, indicating that there is a need for further longitudinal studies before the widespread use of AI in mental health education can be recommended.

Table 4 shows the expanding role of AI in mental health education and professional training. Overall, the results suggest that AI-supported learning settings, simulation methods, and dialogue-based systems could improve educational accessibility, boost participant involvement, and strengthen clinical skill growth. However, there are concerns about data accuracy, the real-world cost of deployment, privacy concerns, and the risk of overreliance on automated solutions. Therefore, careful integration is needed, rather than rapid implementation in these training contexts.

### 3.6. Ethical Governance of Artificial Intelligence in Mental Health

The increased use of AI in mental health care raises ethical, legal, and governance concerns. Although AI technologies may improve diagnostic accuracy, improve accessibility of treatment, support educational outcomes, and increase healthcare efficiency, the reviewed literature also highlighted concerns about possible risks to patients, healthcare professionals, educational institutions, and society in general. Essentially, ethical governance for mental health AI systems is needed so that their development and rollout can be handled in a responsible and systematic manner.

One major ethical area is data privacy and protection. Because AI systems work with sensitive materials, such as healthcare records, behavioral details, speech patterns, social media activity, physiological indications, and data from mobile phones and wearable devices, the stakes are high. Many studies warned about unauthorized access and data breaches, secondary data usage, and cases where users were inadequately informed or had invalid informed consent. Because mental health data are so sensitive, ethical AI adoption would need robust data governance and privacy compliance rather than general assurances. The reviewed literature also focused on algorithmic bias and fairness. The quality and representativeness of the development and training datasets are important determinants of AI model performance. If datasets are biased, the effects may disproportionately affect minority groups, reduce cultural diversity in outcomes, and worsen socioeconomic adversity. Research has shown that biased algorithms can drive higher health inequalities, lower diagnostic accuracy, and even reduce trust in AI-assisted mental health care. In terms of governance measures, the most common approaches were bias audits, fairness assessments, and attempts to create an inclusive dataset from the start.

Transparency and explainability were also key ethical concerns. Complex “black-box” machine learning and deep learning algorithms lead to confusing decisions. Several studies have concluded that the lack of explainability may diminish professional confidence, patient understanding, and responsibility for AI-assisted healthcare judgments. Therefore, explainable AI systems are gaining popularity for transparency, healthcare decision-making, and patient participation. Accountability also emerged as a key challenge. The results raised concerns about the liability of AI systems for misdiagnosis, inappropriate treatment, and harm to patients. Many healthcare systems have questioned legal and professional responsibilities. Several authors called for comprehensive legal frameworks that specify the responsibilities of technology developers, healthcare organizations, physicians, educators, and legislators in the use of AI.

Ethical concerns also extended to governance and education. AI-enabled learning platforms, intelligent tutoring systems, and generative AI tools threaten academic integrity, authenticity of materials, learner dependency, and equitable access to educational resources. Research shows that responsible AI application requires institutional boundaries that protect critical thinking, professional judgment, and quality of training in psychology and psychiatry. The literature suggests that an ethical governance framework is necessary for the application of AI to mental health. Governance should include privacy, fairness, transparency, accountability, safety, and regulation. Without these safeguards, ethical hazards may curtail the potential benefits of AI in public trust, clinical efficacy, and equitable access to mental health care.

Overall, results showed high levels of consensus on the importance of privacy protection, fairness, transparency and accountability as core principles of responsible AI governance in mental health. However, there are still questions to be answered about the most appropriate regulatory models, the legal liability involved in AI-based clinical decisions, and how to implement governance frameworks in different healthcare systems. Most recommendations were based on conceptual analyses, ethical frameworks and policy-oriented publications rather than prospective empirical evaluations, highlighting the need for further implementation research to develop evidence-based governance strategies. The key ethical challenges, their potential consequences, and recommended governance strategies are summarized in Table 5.

The reviewed studies consistently identified privacy, algorithmic bias, transparency, accountability, and safety as the main governance challenges associated with AI use in mental health. Overall, these results stress the need for solid regulatory frameworks so that deployments can be more responsible, dependable, and trustworthy.

## 4. Discussion

Across the three domains, several cross-cutting challenges were consistently identified, including privacy protection, algorithmic bias, transparency, accountability, and limited external validation. However, each domain also demonstrated unique concerns. Clinical applications were primarily limited by validation and implementation barriers; educational applications emphasized AI over-reliance and academic integrity, whereas governance studies focused on regulation, accountability, and ethical oversight.

### 4.1. Interpretation of the Main Results

The present review adopted a domain-based analytical framework to accommodate the multidisciplinary nature of artificial intelligence research in mental health. Clinical applications, educational transformation, and ethical and governance issues were synthesized and interpreted separately before integrating the findings into a unified discussion. This approach enabled a more focused critical interpretation of the available evidence while preserving the comprehensive scope of the review.

This review points to how artificial intelligence (AI) is playing an increasingly important role in mental health and how this influence is evident in clinical routines, professional learning, and governance structures. Although AI tools have achieved substantial progress in several areas, the evidence also indicates that clinical validation, implementation readiness, and regulatory supervision have not kept pace with the rate of technological innovation [32,33,34,35]. A key distinction emerging from this review is that between technical performance and demonstrated clinical utility. A number of studies describe encouraging results for diagnostic accuracy, predictive modeling, and automated decision support, especially for depression, suicide risk, and behavioral monitoring [36,37]. However, much of the evidence appears to come from retrospective data, tightly controlled research settings, or relatively uniform participant groups. Accordingly, strong algorithmic performance under controlled conditions does not necessarily translate into meaningful improvements in patient outcomes or routine clinical effectiveness. Similar concerns, such as the lack of external validation, limited deployment in real-world conditions, and the shortage of long-range follow-up results, appear across the wider digital psychiatry literature [17,23,38,39]. Overall, the results imply that future research should place greater emphasis on clinical utility, implementation readiness, and measurable patient benefits rather than on technological advancement alone.

A comparative analysis of the clinical mental health findings showed agreement and disagreement across the evidence included. There was convergent evidence, in particular, for use in depression and anxiety, suicide-risk assessment, symptom monitoring, and treatment decision support, with several studies suggesting that AI-based approaches may improve pattern detection, risk stratification, accessibility, and clinical decision-making [10,17,20,25,26]. Evidence relating to conversational agents also suggested possible reductions in mild depressive and anxiety symptoms and improved access to psychological support [26]. However, these positive results were not consistent across studies. Differences in the magnitude and clinical robustness of reported benefits were observed, especially in comparisons of the findings of retrospective or internally validated models versus prospective or externally validated studies [20,25].

Some studies reported promising predictive or diagnostic performance, whereas others emphasized limited generalizability, algorithmic bias, false-positive risks, and insufficient evidence in high-risk psychiatric populations. These differences likely reflect heterogeneity in study populations, data sources, AI architectures, outcome definitions, validation procedures, and follow-up periods. More specifically, study-level comparisons illustrate that apparently similar AI applications do not necessarily yield equivalent clinical evidence. For psychiatric diagnosis, Koutsouleris et al. [10] reported promising applications of machine- and deep-learning approaches for early detection and precision-oriented diagnostic support, whereas the broader evidence reviewed here indicates that diagnostic performance varies substantially across populations and models and that external validation remains limited. Chekroud et al. [17] also demonstrated the promise of machine-learning models in predicting treatment response and guiding personalized treatment selection, but other evidence raised issues of model transparency, reproducibility, and generalizability beyond the populations for which the algorithms were trained. In suicide-risk assessment, Garcia de la Garza et al. [23] showed the potential of predictive analytics to identify clinically relevant risk patterns, but this benefit must be interpreted alongside evidence of false-positive risk, algorithmic bias, and limited validation in independent or high-risk populations. A comparable pattern was observed for conversational agents: Abd-Alrazaq et al. [24] reported improvements in depression and anxiety outcomes, whereas the overall evidence remained more cautious regarding sustained effectiveness and the use of such systems in severe or high-risk psychiatric conditions. Similarly, Le Glaz et al. [25] supported the potential of natural-language-processing approaches for symptom detection and monitoring but cross-population validation and data-related bias were still important limitations. Taken together, the study-anchored contrasts suggest that convergence is the strongest around the potential utility of AI, and divergence is primarily in reported benefits in terms of size, reproducibility, external validity, and clinical robustness. The apparent divergence in mental health findings, therefore, does not have to be interpreted as contradictory evidence regarding the value of AI, but rather as an indication that clinical effectiveness is still largely dependent on methodological rigor, external validation, population characteristics and the specific clinical context in which AI is being evaluated [10,25,26].

This disconnect illustrates the “AI implementation gap,” a term increasingly used to describe the disconnect between promising results of research algorithms and successful translation into routine clinical practice. Several factors may contribute to this gap, including lack of generalizability of training data, potential for model overfitting, fragmentation of electronic health record systems, heterogeneity of healthcare infrastructure, and organizational barriers to implementation. From an implementation science perspective, successful integration of AI into mental health care requires not only technically sound algorithms but also clinician buy-in, workflow integration, prospective real-world evaluation, and ongoing monitoring to ensure that technological advances lead to meaningful improvements in patient care. Beyond clinical applications, AI is increasingly being incorporated into professional education and workforce development. However, evidence regarding its long-term educational effectiveness and impact on professional competencies remains limited [10,17,23].

Finally, our findings underscore the central role of governance in shaping the future of AI adoption. Across clinical, educational, and organizational settings, recurring concerns include privacy, algorithmic bias, transparency, accountability, and safety [7,16,29,40].

Rather than representing isolated ethical issues, these factors appear to function as structural determinants of successful implementation. Consequently, the long-term value of AI in mental health will depend not only on technological capability but also on the effectiveness of governance frameworks in ensuring equitable, safe, and trustworthy deployment. Table 6 summarizes the principal areas of convergence and divergence across AI application domains, together with the methodological and contextual factors that may explain inconsistent findings.

### 4.2. Clinical, Educational and Governance Implications

An important implication of the present findings is that the successful integration of AI into mental health systems requires a broader perspective than technological performance alone. Sustainable implementation depends on organizational readiness, workforce competencies, ethical oversight, and institutional trust. Accordingly, healthcare organizations, educational institutions, and policymakers should approach AI adoption as a socio-technical transformation rather than a purely technological innovation, consistent with principles of human-centered and responsible AI [7,19].

The findings support a human-centered approach to AI integration in mental health. Although AI can enhance early detection, risk assessment, and treatment planning, its greatest value appears to lie in augmenting rather than replacing professional expertise [32,48]. Therefore, future evaluation frameworks should extend beyond predictive performance and include clinical utility, patient acceptability, and implementation feasibility as key indicators of success.

In education, AI provides some opportunities for tailored learning, simulation training, and ongoing professional growth, but it should complement human instruction and mentorship rather than replace them. The review also suggests that ethical and regulatory considerations have become core requirements if the use is meant to keep working in a sustainable way. Without proper guardrails for privacy, fairness, transparency, and accountability, even technically successful AI systems may have difficulty earning clinical acceptance and building public trust [1].

### 4.3. Interrelationships Among Clinical, Educational, and Governance Domains

The findings from this review highlight the interrelated nature of the clinical, educational and governance aspects of AI implementation rather than separate domains. Progress in clinical applications relies not only on the performance of algorithms, but also on the preparedness of healthcare professionals to interpret and use AI-supported recommendations appropriately. Therefore, educational programs are key to developing AI literacy, critical appraisal skills and responsible clinical decision-making. At the same time, effective governance frameworks provide the ethical and regulatory foundations needed to support both the safe clinical implementation and the responsible educational use of AI technologies. Similarly, challenges identified in one domain frequently impact on the others. For example, challenges and issues of transparency and explainability can erode clinician trust, training and regulatory accountability, while privacy, algorithmic bias and fairness can affect clinical effectiveness, educational acceptability and public trust simultaneously. These interdependencies show that successful integration of AI into mental health systems requires parallel advances in clinical practice, professional education and governance, rather than isolated technological progress.

### 4.4. Future Directions and Emerging Challenges

A major priority for future research is the rapid development of generative artificial intelligence and large language models (LLMs). Even if these tools have shown real promise in messaging, information retrieval, psychoeducation, and decision-support tasks, their actual use in mental health still has considerable uncertainty attached to it. Previous studies have raised concerns about hallucinated outputs, uneven or contradictory suggestions, insufficient explainability, and the genuine risk of delivering clinically incorrect, or even potentially harmful, information. These issues are particularly important in mental health contexts, where individuals may seek support during periods of emotional vulnerability requiring timely assistance. Therefore, future research should not only linger at the technical performance level, but also place more emphasis on safety, reliability, transparency, and real-world clinical utility. This will require evidence-based standards for validation, continuous monitoring, and regulatory governance, so the responsible integration into mental healthcare systems is supported in practice, and not simply assumed [16,49,50,51,52].

Future research and implementation also involves (1) agile regulatory frameworks that can respond rapidly to the fast evolution of AI technologies and balance regulatory agility and patient safety and public trust; (2) fairness, inclusiveness, and digital divide mitigation to ensure equitable access across different populations and healthcare settings; and (3) a paradigm shift from retrospective algorithm development to prospective, real-world clinical evaluations that assess patient outcomes, implementation feasibility, and long-term effectiveness. Fourth, future research should define optimal models of human–AI collaboration that outline complementary roles of clinicians and AI systems in decision-making. Finally, interdisciplinary education and AI literacy should be part of professional training to ensure that healthcare practitioners are equipped to critically evaluate, safely implement and responsibly govern AI technologies in mental health practice.

### 4.5. Theoretical and Practical Contributions

This review helps add to the growing literature on artificial intelligence in mental health by offering an integrated view that examines clinical uses, changes in education, and ethical governance at the same time. Much earlier work has tended to focus on one technical piece at a time, such as machine learning models, conversational agents, or digital mental health interventions considered separately. This review highlights how technological innovation, workforce development, and governance capability connect together and influence whether AI is actually adopted well in mental health systems [32,52]. On the theoretical side, the results strengthen the idea that AI’s value in mental healthcare is greatest when computational power is tied with human expertise, ethical reasoning, and care that stays patient-centered. This is in line with current conversations stating that AI should not replace clinical judgment, the therapeutic bond, and how clinicians make professional decisions [34]. The review also suggests that any future theoretical model of AI-enabled mental healthcare should include more than just technical performance measures. They should also cover educational preparedness, organizational readiness, and actual governance mechanisms as core drivers of success.

Practically, the findings point toward workforce training, interdisciplinary collaboration, regulatory oversight, and ongoing assessment of AI-enabled mental health systems. Sustainable deployment will hinge on keeping technological innovation in step with clinical validity, educational quality, ethical accountability, and public trust [35,41].

## 5. Strengths and Limitations

This systematic review presents a consolidated synthesis of the current evidence on artificial intelligence in mental health by examining clinical applications, educational transformation, and ethical and governance issues within a single analytical framework. The review benefited from methodological transparency and comprehensiveness through a structured domain-based synthesis, adherence to PRISMA 2020 recommendations, and the inclusion of multidisciplinary evidence. More importantly, the comparative synthesis of convergent and divergent findings allowed a more critical interpretation of the consistency, clinical relevance and methodological variability of the evidence available.

Several limitations should, however, be considered when interpreting the findings. First, substantial heterogeneity was observed across study designs, participant populations, AI methodologies, outcome measures, and validation procedures. This heterogeneity limited direct comparability across studies and precluded a meaningful quantitative meta-analysis, thereby necessitating a structured narrative synthesis. Second, a large proportion of the available evidence came from pilot studies, retrospective datasets, or controlled research settings, and relatively few studies involved rigorous external validation or prospective multicenter clinical evaluation. Thus, the degree to which the reported benefits are translated into routine clinical practice across different populations and healthcare systems is uncertain.

Third, the rapid evolution of artificial intelligence, particularly large language models and generative AI, means that the evidence base is continuously changing. The findings of this review should therefore be interpreted according to the literature available at the time of the predefined search period and continued evaluation of clinical effectiveness, safety and ethical implications will be required as new evidence becomes available. Fourth, the review protocol was not prospectively registered with PROSPERO or another publicly accessible registry. Although a predefined protocol was followed and the review was conducted in accordance with the recommendations of PRISMA 2020, prospective registration would have increased methodological transparency and reduced the potential risk of reporting bias. Finally, the review was limited to English-language peer-reviewed publications, which may have resulted in the exclusion of relevant evidence published in other languages or outside the peer-reviewed literature.

Despite these limitations, the findings provide a structured overview of the current evidence and highlight the need for future research emphasizing prospective evaluation, external validation, methodological standardization, and real-world clinical implementation before widespread integration of AI into routine mental health practice can be recommended.

## 6. Conclusions

Artificial Intelligence (AI) is increasingly transforming the practice, education and governance of mental health care. The evidence synthesized in this review indicates that AI has the potential to support diagnostic decision-making, early intervention, access to mental health services, education, and clinical decision support. However, current evidence must be treated with caution as many AI applications remain at an experimental stage, with limited external validation and no consistent generalisability across different clinical populations and healthcare settings. Importantly, although convergent findings support the potential value of AI across several mental health applications, divergent findings indicate that the magnitude and clinical robustness of these benefits vary according to study design, population characteristics, validation procedures, and clinical context.

Persistent challenges such as privacy protection, algorithmic bias, transparency, accountability and the complexity of real-world implementation continue to limit routine clinical adoption. Therefore, AI should be regarded as a complementary tool rather than a replacement for human expertise. Future progress will depend not only on interdisciplinary collaboration and robust governance frameworks but also on rigorous prospective clinical evaluation, external validation, transparent algorithm development, and continuous ethical oversight before AI can be routinely integrated into mental health practice.

## Figures and Tables

**Table 1:** Databases and Search Strategy.

Database	Coverage Area	Search Period	Search Date	Search Filters	Search Strategy
Scopus	Multidisciplinary scientific literature	January 2019–December 2025	31 December 2025	English language, peer-reviewed articles, publication years (2019–2025)	Database-specific strategy adapted to Scopus indexing (see Supplementary Material S1).
Web of Science Core Collection	Multidisciplinary scientific literature	January 2019–December 2025	31 December 2025	English language, peer-reviewed articles, publication years (2019–2025)	Database-specific strategy adapted to Web of Science indexing (see Supplementary Material S1).
PubMed/MEDLINE	Medicine, Psychiatry, Mental Health	January 2019–December 2025	31 December 2025	English language, human studies where applicable, and publication years (2019–2025)	Database-specific strategy using MeSH terms and free-text keywords (see Supplementary Material S1).
PsycINFO	Psychology and Behavioral Sciences	January 2019–December 2025	31 December 2025	English language, peer-reviewed publications, publication years (2019–2025)	Database-specific strategy adapted to PsycINFO indexing (see Supplementary Material S1).
Google Scholar	Multidisciplinary scholarly literature	January 2019–December 2025	31 December 2025	First 200 records ranked by relevance; English-language publications This approach has been widely adopted in systematic reviews because of the large volume and ranking algorithm of Google Scholar.	Database-specific strategy adapted for Google Scholar (see Supplementary Material S1).

**Table 2:** Characteristics of the Included Studies (*n* = 88).

Characteristic	Category	Frequency (*n*)	Percentage (%)
Publication Year	2019	2	2.3
	2020	14	15.9
	2021	18	20.5
	2022	14	15.9
	2023	14	15.9
	2024	12	13.6
	2025	14	15.9
Study Design	Systematic Reviews	18	20.5
	Randomized Controlled Trials	11	12.5
	Observational Studies	19	21.6
	Cross-sectional Studies	15	17.0
	Mixed Methods Studies	10	11.4
	Technology Development Studies	15	17.0
Geographic Region	North America	31	35.2
	Europe	24	27.3
	Asia	20	22.7
	Oceania	8	9.1
	Middle East and Africa	5	5.7
AI Technology	Machine Learning	34	38.6
	Deep Learning	19	21.6
	Natural Language Processing	13	14.8
	Conversational Agents/Chatbots	14	15.9
	Large Language Models	8	9.1
Mental Health Domain	Depression and Anxiety	32	36.4
	Suicide Risk Assessment	12	13.6
	Stress and Well-being	15	17.0
	Severe Mental Disorders	11	12.5
	General Mental Health Applications	18	20.5

Note: The categories presented in Table 2 are not mutually exclusive. Studies applying multiple AI techniques or addressing multiple mental health conditions were classified according to their primary focus for descriptive purposes, whereas secondary characteristics were incorporated into the narrative synthesis.

**Table 3:** Clinical applications of artificial intelligence in mental health.

Clinical Application	Representative Study	AI Technology	Primary Objective	Reported Benefits	Key Challenges
Mental Health Diagnosis	Koutsouleris et al. (2022) [10]	Machine Learning, Deep Learning	Early detection and diagnostic support for psychiatric disorders	Potential support for precision psychiatry and diagnostic decision-making	Clinical implementation barriers and limited generalizability
Suicide Risk Prediction	Garcia de la Garza et al. (2021) [23]	Machine Learning, Predictive Analytics	Identification of individuals at elevated suicide risk	Improved identification of relevant risk patterns	Algorithmic bias, false positives, and ethical concerns
Treatment Decision Support	Chekroud et al. (2021) [17]	Machine Learning Models	Prediction of treatment response and personalized treatment selection	Potential improvement in treatment-response prediction	Transparency and external validation requirements
Conversational Agents and Chatbots	Abd-Alrazaq et al. (2020) [24]	NLP, Conversational Agents, Chatbots	Delivery of psychological support and psychoeducation	Reported improvements in depression and anxiety outcomes	Safety concerns and limited evidence for high-risk clinical situations
Symptom Monitoring	Le Glaz et al. (2021) [25]	Natural Language Processing	Detection and monitoring of mental states and psychiatric symptoms	Potentially accurate symptom monitoring using language-derived data	Data bias and limited cross-population validation
Relapse Prediction	—	Predictive Analytics	Forecasting symptom deterioration and relapse	Potential for proactive clinical management	Limited longitudinal and externally validated evidence

**Table 4:** Applications of AI in mental health education and professional training.

Educational Application	Representative Study (Example)	AI Technology	Educational Purpose	Reported Benefits	Education-Specific Challenges
Adaptive Learning Systems	Ouyang et al. (2022) [20]	Machine Learning	Personalized education	Improved learner engagement and adaptive learning	Dependence on algorithm-generated learning pathways and reduced learner autonomy
Virtual Patients	Rangel-de Lazaro & Duart (2023) [26]	AI-Simulation/Extended Reality	Clinical skills training	Enhanced clinical reasoning and practical competency	Limited realism and inability to fully replicate complex patient interactions
Intelligent Tutoring Systems	Tozsin et al. (2024) [27]	Machine Learning	Individualized feedback	Improved learning efficiency and personalized instruction	Over-reliance on automated feedback and reduced critical thinking
Conversational AI Assistants	Kasneci et al. (2023) [16]	NLP, Large Language Models	Educational support	Immediate access to educational resources and self-directed learning	AI hallucinations, inaccurate educational content, and academic integrity concerns
Predictive Learning Analytics	Garzon et al. (2025) [28]	Machine Learning	Student performance monitoring	Early identification of learning difficulties and personalized educational support	Student privacy, algorithmic bias, and ethical concerns regarding learner evaluation

**Table 5:** Ethical challenges and governance issues of AI-enabled mental health systems.

Ethical Domain	Representative Study	Key Challenge	Potential Consequences	Recommended Governance Strategy
Privacy and Data Protection	Fiske et al. (2020) [29]	Sensitive mental health data exposure	Loss of confidentiality and reduced public trust	Robust data governance, encryption, and secure data management
Algorithmic Bias	Walsh et al. (2020) [7]	Unequal model performance across populations	Healthcare disparities and discriminatory clinical decisions	Bias auditing, representative datasets, and fairness monitoring
Transparency and Explainability	Gooding & Kariotis (2021) [1]	Black-box decision-making	Reduced clinician trust and limited interpretability	Explainable AI frameworks and transparent model reporting
Accountability	Reddy et al. (2020) [30]	Unclear responsibility for AI-assisted decisions	Legal uncertainty and ethical ambiguity	Clear regulatory policies and professional accountability frameworks
Safety and Reliability	Saeidnia et al. (2024) [31]	Inaccurate predictions or recommendations	Patient harm and reduced clinical effectiveness	Prospective clinical validation and continuous performance monitoring
Educational Ethics	Kasneci et al. (2023) [16]	Over-reliance on AI-generated educational content	Reduced critical thinking and threats to academic integrity	Institutional AI governance policies, faculty oversight, and AI literacy training

**Table 6:** Comparative synthesis of convergent and divergent findings across AI applications in mental health.

Application Area	Convergent Findings	Divergent Findings	Main Sources of Divergence	Representative Evidence
Diagnosis & early detection	ML/DL approaches show consistent potential for detecting depression, anxiety, psychosis, and bipolar disorder using EHR, neuroimaging, speech, and behavioral data.	Performance varies across populations and models, with limited external or prospective validation.	Study design, sample characteristics, data modality, case definitions, and validation strategy.	[5,10,38,41,42,43]
Suicide-risk prediction	AI models support large-scale risk stratification and identification of individuals at elevated risk.	False-positive rates, subgroup performance, and limited validation in high-risk populations remain concerns.	Outcome definition, base-rate differences, non-representative training data, and demographic heterogeneity.	[7,22]
Treatment-response prediction	ML shows promise for predicting treatment response and supporting personalized treatment selection.	Performance and reproducibility may decline across independent or externally validated cohorts.	Overfitting, site-specific data, outcome definitions, validation strategy, and follow-up duration.	[16,24]
Conversational agents & chatbots	Evidence suggests short-term improvement in mild depression/anxiety symptoms and improved accessibility to psychological support.	Effects are heterogeneous, with limited evidence for sustained benefit or use in severe/high-risk conditions.	Follow-up duration, symptom severity, intervention design, and adjunctive versus standalone use.	[15,23,44,45]
Symptom monitoring & digital phenotyping	NLP and sensor-based approaches show potential for monitoring mood, sleep, and other mental-state indicators.	Evidence remains predominantly observational, with limited longitudinal and cross-population validation.	Small or homogeneous samples, platform variability, missing data, and data-related bias.	[24,42]
Education & training	AI-supported adaptive learning, virtual patients, and tutoring may improve engagement, clinical reasoning, and access to training.	Long-term competency transfer and effects on critical thinking remain uncertain.	Short follow-up, heterogeneous learners, and variation in educational outcomes.	[15,19,25,26,46]
Ethics & governance	Strong agreement exists on the importance of privacy, fairness, transparency, accountability, and safety.	Consensus is limited regarding regulatory models, liability, and cross-system implementation.	Jurisdictional differences, limited empirical testing, and rapidly evolving AI technologies.	[1,7,28,29,47]

## Data Availability

No new data were created or analyzed in this study. Data sharing is not applicable to this article.

## Supplementary Materials

The following supporting information can be downloaded at: https://www.mdpi.com/article/10.3390/healthcare14172721/s1, S1: Database-Specific Search Strategies; S2: Methodological Quality Assessment of the Included Studies.

## Appendix A. Study Characteristics, AI Applications, Key Contributions, and Main Challenges of the Included Studies

**Table A1:** Study Characteristics, AI Applications, Key Contributions, and Main Challenges of the Included Studies.

Authors (Ref.)	Domain	Study Design	AI Technology/Approach	Application Area	Key Contribution/Focus	Key Challenges/Concerns
Abd-Alrazaq et al. (2020) [24]	CP	Systematic Review	Chatbots	Mental health support	Improved depression and anxiety outcomes	Safety concerns
D’Alfonso (2020) [38]	CP	Review	Artificial Intelligence	Mental healthcare delivery	Improved access to services	Privacy concerns
Fernandes et al. (2020) [39]	CP	Review	Machine Learning	Precision psychiatry	Enhanced diagnostic potential	Validity concerns
Le Glaz et al. (2021) [25]	CP	Systematic Review	Natural Language Processing	Mental state detection	Accurate symptom monitoring	Data bias
Chekroud et al. (2021) [17]	CP	Machine Learning Study	Machine Learning	Treatment prediction	Improved treatment response prediction	Transparency issues
Garcia de la Garza et al. (2021) [23]	CP	Review	Machine Learning	Suicide risk prediction	Effective risk identification	Algorithmic bias
Koutsouleris et al. (2022) [10]	CP	Review	Machine Learning	Psychiatric diagnosis	Supported precision psychiatry	Clinical implementation barriers
Sun et al. (2023) [44]	CP	Review	Artificial Intelligence	Psychiatric applications	Improved diagnostic performance	Governance requirements
Zhong et al. (2024) [53]	CP	Systematic Review and Meta-Analysis	AI-Based Chatbots	Depressive and anxiety symptoms	Evaluated the short-term therapeutic effectiveness of AI chatbots	Heterogeneity, short follow-up, and limited long-term evidence
Casu et al. (2024) [54]	CP	Review	Artificial Intelligence	Mental health interventions	Positive intervention outcomes	Ethical oversight
Dehbozorgi et al. (2025) [45]	CP	Systematic Review	Artificial Intelligence	Mental health applications	Broad effectiveness across domains	Implementation challenges
Omarov et al. (2023) [47]	CP	Systematic Review	Chatbots	Mental healthcare	Promising clinical support	Safety and trust
Wang et al. (2025) [55]	EG	Systematic Review	Generative AI	Mental health support	Expanding applications	Ethical implications
Fanarioti & Karpouzis (2025) [56]	CP	Review	Artificial Intelligence	Future mental healthcare	Digital transformation opportunities	Governance concerns
Wimbarti et al. (2024) [57]	CP	Review	Artificial Intelligence	Self-diagnosis in mental health	Potential for screening	Risk of misuse
Ni & Jia (2025) [42]	CP	Scoping Review	Artificial Intelligence	Digital mental health interventions	Broad intervention coverage	Responsible implementation
Blease et al. (2019) [32]	CP	Review	Digital AI	Primary mental healthcare	Increased accessibility	Trust concerns
Birnbaum et al. (2020) [58]	CP	Feasibility Study/Original Research	Artificial Intelligence	Early psychosis detection	Early identification benefits	Privacy concerns
Bracher-Smith et al. (2021) [5]	CP	Review	Machine Learning	Mental health prediction	Strong predictive potential	Bias concerns
Brown & Halpern (2021) [59]	CP	Review	Artificial Intelligence	Empathy and mental health	Human–AI interaction insights	Ethical considerations
Buchanan et al. (2021) [36]	ET	Scoping Review	Artificial Intelligence	Nursing education	AI may transform nursing education	Educational preparedness
Charow et al. (2021) [6]	ET	Scoping Review	Artificial Intelligence	Health professional education	Need for AI competencies	Ethical literacy
Ouyang et al. (2022) [20]	ET	Systematic Review	Artificial Intelligence	Higher education	Improved learning outcomes	Data privacy
Gray et al. (2022) [60]	ET	Review	Artificial Intelligence	Health workforce education	Skills gap identified	Governance awareness
Mir et al. (2023) [61]	ET	Review	Artificial Intelligence	Medical education	Curriculum innovation	Responsible use
Kasneci et al. (2023) [16]	ET	Review	Generative AI	Higher education	Educational opportunities	Academic integrity
Forero-Corba & Bennasar (2024) [62]	ET	Systematic Review	AI/Machine Learning	Education	Improved educational outcomes	Transparency concerns
Tozsin et al. (2024) [27]	ET	Systematic Review	Artificial Intelligence	Medical education	Enhanced training effectiveness	Ethical implementation
Hallquist et al. (2025) [63]	ET	Systematic Review	Artificial Intelligence	Medical education	Improved assessment and teaching	Governance needs
Shishehgar et al. (2025) [46]	ET	Systematic Review	Artificial Intelligence	Health education	Positive student perceptions	Ethical concerns
Luo et al. (2025) [64]	ET	Systematic Review	Artificial Intelligence	Medical student readiness	Moderate readiness for AI integration among medical students	Anxiety concerns
Garzon et al. (2025) [28]	ET	Systematic Review	Artificial Intelligence	Education systems	Identified benefits and challenges of AI integration	Responsible implementation
Arar et al. (2025) [65]	ET	Systematic Review	Artificial Intelligence	Educational leadership	AI supports educational management and leadership	Governance requirements
Rangel-de Lazaro & Duart (2023) [26]	ET	Systematic Review	AI/Extended Reality (XR)	Online education	Improved student engagement	Ethical deployment
Gado et al. (2022) [43]	CP	Review	Artificial Intelligence	Mental health practice	Expanded clinical applications of AI	Ethical oversight
Parmigiani et al. (2022) [66]	CP	Systematic Review	Artificial Intelligence	Clinical decision support	Improved clinical decision-making	Accountability concerns
Rogan et al. (2024) [67]	CP	Systematic Review with Meta-Synthesis	Passive Sensing, AI, Machine Learning	Mental health monitoring	Identified implementation facilitators and barriers from clinicians’ perspectives	Data privacy, clinician acceptance, workflow integration, governance
Gooding & Kariotis (2021) [1]	EG	Scoping Review	Artificial Intelligence	Mental health regulation	Identified legal and ethical gaps	Governance framework needed
Ienca & Ignatiadis (2020) [34]	EG	Review	Artificial Intelligence	Clinical neuroscience	Documented ethical challenges	Accountability concerns
Walsh et al. (2020) [7]	EG	Review	Algorithms	Algorithmic decision-making	Identified bias as a major risk	Fairness concerns
Fiske et al. (2020) [29]	EG	Review	Artificial Intelligence	Healthcare ethics	Proposed ethical principles for AI	Governance and trust
Jacobson et al. (2020) [68]	EG	Review	Artificial Intelligence	Mental healthcare	Highlighted ethical dilemmas	Transparency concerns
Reddy et al. (2020) [30]	EG	Governance Review	Artificial Intelligence	Healthcare governance	Proposed governance model	Regulatory oversight
Gerke et al. (2020) [69]	EG	Review	Artificial Intelligence	AI-driven healthcare	Identified legal and ethical risks	Liability issues
Char et al. (2020) [70]	EG	Review	Machine Learning	Healthcare applications	Recommended ethical safeguards	Responsible deployment
Mörch et al. (2020) [71]	EG	Framework Study	Artificial Intelligence	Suicide prevention	Proposed ethical checklist	Governance standards
Straw & Callison-Burch (2020) [72]	EG	Review	Artificial Intelligence	Algorithmic fairness	Recommended bias mitigation strategies	Equity concerns
Crossnohere et al. (2022) [73]	EG	Literature Review and Content Analysis	Artificial Intelligence	AI governance frameworks	Frameworks support responsible AI implementation	Ethical compliance
Prakash et al. (2022) [74]	EG	Scoping Review	Artificial Intelligence	Healthcare ethics	Identified multiple ethical challenges	Governance gaps
Rubeis (2022) [75]	EG	Review	AI & Big Data	Mental healthcare	Discussed ethical implications of intelligent health systems	Privacy and autonomy
Salah et al. (2024) [76]	EG	Review	GPT/Large Language Models	Cognitive and mental health impacts	Identified emerging opportunities and risks	Ethical governance required
Tavory (2024) [77]	EG	Review	Artificial Intelligence	Mental health regulation	Proposed an ethics-of-care perspective	Regulatory oversight
Saeidnia et al. (2024) [31]	EG	Review	Artificial Intelligence	Mental health interventions	Emphasized responsible implementation	Ethical safeguards
Ortega-Bolaños et al. (2024) [78]	EG	Systematic Review	Artificial Intelligence	Ethical assessment tools	Identified frameworks for AI evaluation	Responsible AI development
Batool et al. (2025) [79]	EG	Systematic Review	Artificial Intelligence	AI governance	Synthesized governance mechanisms	Responsible AI principles
Robles & Mallinson (2025) [80]	EG	Systematic Review	Artificial Intelligence	Public governance	Proposed unified governance framework	Accountability
Ismail & Ahmad (2025) [81]	EG	Systematic Review	Artificial Intelligence	Ethical governance frameworks	Identified comprehensive governance models	Ethical implementation
Blease & Rodman (2025) [48]	EG	Ethical Review	Generative AI	Mental healthcare	Discussed ethical implications of generative AI	Patient trust and safety
Abusamra et al. (2025) [82]	EG	Systematic Review	Artificial Intelligence	Pediatric medicine	Identified ethical and practical implications	Governance requirements
Vilaza & McCashin (2021) [83]	EG	Review	AI Chatbots	CBT and mental health	Evaluated chatbot-supported CBT	Governance and accountability
Murphy et al. (2021) [84]	EG	Scoping Review	Artificial Intelligence	Healthcare ethics	Identified broad ethical themes in AI-enabled healthcare	Equity and justice
Yangi et al. (2025) [85]	EG	Review	ChatGPT	Medicine and healthcare	Summarized benefits and limitations of ChatGPT	Ethical risk management
Chandler (2020) [86]	CP	Review	Artificial Intelligence	Mental healthcare innovation	AI supports transformation of mental health services	Ethical oversight
Boucher et al. (2021) [4]	CP	Review	Chatbots	Digital mental health interventions	Positive outcomes of chatbot-based interventions	Privacy concerns
Shatte et al. (2019) [87]	CP	Systematic Review	Machine Learning	Mental health prediction	Demonstrated predictive capability of ML models	Bias concerns
Mohr et al. (2021) [40]	CP	Review	Digital Mental Health	Treatment delivery	Technology supports mental healthcare pathways	Ethical implementation
Singhal et al. (2023) [51]	CP	Review	Large Language Models	Mental healthcare support	Described potential applications of LLMs in assessment and support	Validation and implementation challenges
Chivilgina et al. (2020) [88]	CP	Systematic Review	mHealth & Artificial Intelligence	Schizophrenia management	Mobile AI tools improve monitoring	Privacy concerns
Chivilgina et al. (2021) [89]	CP	Review	Digital Technologies	Schizophrenia care	Improved disease management	Ethical considerations
Graham et al. (2025) [9]	EG	Commentary Review	Digital Mental Health	Service advocacy	Emphasized equity and accessibility	Safe implementation
Jermutus et al. (2022) [90]	CP	Review	Artificial Intelligence	Mental health analytics	Improved symptom monitoring	Data governance
Zhou et al. (2022) [91]	CP	Review	Artificial Intelligence	Psychological diagnosis and intervention	Promising clinical applications	Ethical deployment
Torous et al. (2021) [3]	CP	Review	Digital AI	Digital psychiatry	Expanded access and continuous monitoring	Regulation and governance
Coghlan et al. (2023) [92]	EG	Review	AI Chatbots	Mental health chatbots	Examined ethical implications of chatbot use	Trust, safety, and accountability
Demszky et al. (2023) [93]	CP	Review	Large Language Models	Psychology and mental health research	LLMs support psychological assessment and research	Validity and reliability concerns
Denecke et al. (2021) [94]	CP	Usability Study	Chatbot	Emotional regulation support	Positive usability and emotional support	User engagement and safety
Garcia-Martínez et al. (2023) [23]	CP	Review	Artificial Intelligence	Mental healthcare applications	Expanded opportunities for AI-enabled care	Implementation challenges
Garg et al. (2023) [95]	CP	Systematic Review	ChatGPT	Clinical care and medical research	Potential applications in diagnosis and treatment	Accuracy and ethical concerns
Garriga et al. (2022) [96]	CP	Original Research/Prospective Validation Study	AI & Predictive Analytics	Psychiatry and mental healthcare	Improved predictive decision-making	Data governance concerns
Chelli et al. (2025) [97]	CP	Review	ChatGPT (Hallucination & Reference Accuracy)	Mental health applications	Evaluated potential of LLMs in mental healthcare	Bias and responsible use
Lee et al. (2021) [2]	CP	Review	Artificial Intelligence	Mental healthcare delivery	AI supports diagnosis and clinical decision-making	Clinical implementation barriers
Lomis et al. (2021) [98]	ET	Perspective/Expert Commentary	Artificial Intelligence	Health professions education	Highlighted need for AI literacy	Curriculum integration challenges
Malgaroli et al. (2023) [99]	CP	Review	Artificial Intelligence	Digital mental health	Expanded opportunities for digital mental healthcare	Evidence and governance concerns
Mesko & Topol (2023) [50]	EG	Review	Large Language Models	Healthcare governance	Highlighted need for regulatory oversight of LLMs	Accountability and safety
Obradovich et al. (2024) [100]	CP	Review	Large Language Models	Psychiatry	Identified opportunities for AI-assisted psychiatric care	Risk management requirements
Panesar (2023) [101]	CP	Review	Artificial Intelligence & Machine Learning	Precision mental health diagnostics	Enhanced diagnostic and predictive capabilities	Model transparency concerns
Scholich et al. (2025) [102]	CP	Comparative Study	Chatbots	Mental health support	Compared therapists with AI chatbots	Trust and effectiveness concerns
Welch et al. (2022) [18]	CP	Scoping Review	Mobile AI & Wearables	Child and adolescent psychiatry	Potential for monitoring and intervention	Privacy and ethical concerns

Abbreviations: AI, Artificial Intelligence; CP, Clinical Practice; ET, Educational Transformation and Professional Training; EG, Ethical, Legal, and Governance; LLMs, Large Language Models; NLP, Natural Language Processing.
