# Supplementary material for: Artificial Intelligence Applications in Mental Health: A Systematic Review of Clinical Practice, Educational Transformation, and Ethical Governance
Source: Healthcare (Basel). 2026 Aug 26;14(17):2721. doi: 10.3390/healthcare14172721 (PMC13565381; doi:10.3390/healthcare14172721)
Supplement: Supplementary file 1 [file healthcare-14-02721-s001.zip › healthcare-4427453-supplementary-S2.pdf]

Supplementary Material S2

Table S2. Verified methodological appraisal framework for included studies

Methodological note: Study designs and appraisal tools were verified against the original publications. Where verification resulted in changes to the study design or appraisal instrument, the methodological appraisal was updated accordingly using the appropriate critical appraisal tool. This process was undertaken to ensure consistency, methodological accuracy, and transparency across all included studies.

No.	First Author (Year)	Verified Study Design	Appraisal Tool	Overall Appraisal	Main Methodological Limitation / Action
1	Abd-Alrazaq et al. (2020)	Systematic Review	AMSTAR 2	High	Considerable heterogeneity among included studies and limited evidence for high-risk clinical settings.
2	D'Alfonso (2020)	Narrative Review	SANRA	Moderate	Narrative design without systematic study selection or formal risk-of-bias assessment.
3	Fernandes et al. (2020)	Diagnostic Machine-Learning Study	PROBAST	**Moderate**	Internal validation only; no independent external validation and possible spectrum bias due to case-control design.
4	Le Glaz et al. (2021)	Systematic Review	AMSTAR 2	High	Variability in NLP methodologies and outcome measures limited direct comparisons.
5	Chekroud et al. (2021)	Narrative Review	SANRA	**High**	Review-level synthesis with limited prospective and external evidence for clinical treatment selection.
6	Garcia de la Garza et al. (2021)	Longitudinal Predictive Modelling Study	PROBAST	**Moderate**	Model was cross-validated within one national dataset but lacked independent external validation.
7	Koutsouleris et al. (2022)	Narrative Review	SANRA	**Moderate**	Comprehensive conceptual synthesis of precision psychiatry; recommendations remain largely unsupported by prospective clinical implementation studies.
8	Sun et al. (2023)	Narrative Review	SANRA	Moderate	Limited discussion of methodological quality and external validation across included studies.
9	Zhong et al. (2024)	Systematic Review and Meta-Analysis	AMSTAR 2	**High**	Short-term pooled effects were promising, but heterogeneity and limited long-term follow-up constrained certainty.
10	Casu et al. (2024)	Scoping Review	JBI Checklist for Systematic Reviews and Research Syntheses	**Moderate**	Evidence mapping identified heterogeneous applications with limited comparative and long-term clinical validation.
11	Dehbozorgi et al. (2025)	Systematic Review	AMSTAR 2	High	Considerable heterogeneity in AI applications and study methodologies.
12	Omarov et al. (2023)	Systematic Review	AMSTAR 2	High	Limited long-term clinical evidence and substantial heterogeneity among chatbot interventions.
13	Wang et al. (2025)	Systematic Review	AMSTAR 2	Moderate	Heterogeneity of generative-AI applications and limited empirical governance evidence.
14	Fanarioti & Karpouzis (2025)	Narrative Review	SANRA	Moderate	Broad future-oriented synthesis with limited systematic appraisal of the evidence.
15	Wimbarti et al. (2024)	Narrative Review	SANRA	Moderate	Limited empirical validation of AI-supported self-diagnosis and potential selection bias.
16	Ni & Jia (2025)	Scoping Review	JBI Checklist for Systematic Reviews and Research Syntheses	High	Broad mapping approach with limited ability to determine intervention effectiveness.
17	Blease et al. (2019)	Cross-Sectional Survey Study	JBI Analytical Cross-Sectional Checklist	**Moderate**	Perception-based survey findings do not establish clinical effectiveness or replacement of professional roles.
18	Birnbaum et al. (2020)	Predictive Digital-Phenotyping Study	PROBAST	**Moderate**	Small selected sample and lack of independent external validation limited generalizability.
19	Bracher-Smith et al. (2021)	Systematic Review	AMSTAR 2	High	Heterogeneity in datasets, genetic predictors, and model-validation approaches.
20	Brown & Halpern (2021)	Ethical/Conceptual Article	JBI Checklist for Text and Opinion Papers	**Moderate**	Conceptual ethical argument without empirical testing of clinical or relational outcomes.
21	Buchanan et al. (2021)	Scoping Review	JBI Checklist for Systematic Reviews and Research Syntheses	Moderate	Broad evidence mapping and limited evaluation of educational effectiveness.
22	Charow et al. (2021)	Scoping Review	JBI Checklist for Systematic Reviews and Research Syntheses	High	Limited evidence concerning long-term competency development and implementation outcomes.
23	Ouyang et al. (2022)	Systematic Review	AMSTAR 2	High	Heterogeneity in educational settings, technologies, and learning outcomes.

No.	First Author (Year)	Verified Study Design	Appraisal Tool	Overall Appraisal	Main Methodological Limitation / Action
24	Gray et al. (2022)	Expert Survey Study	JBI Analytical Cross-Sectional Checklist	Moderate	Expert-opinion data described educational needs but did not evaluate learner or clinical outcomes.
25	Mir et al. (2023)	Narrative Review	SANRA	Moderate	Limited systematic search information and predominantly descriptive evidence.
26	Kasneci et al. (2023)	Position Paper / Commentary	JBI Checklist for Text and Opinion Papers	Moderate	Conceptual analysis of LLM opportunities and risks without direct testing of educational effectiveness.
27	Forero-Corba & Bennasar (2024)	Systematic Review	AMSTAR 2	High	Substantial heterogeneity in AI techniques, educational contexts, and outcomes.
28	Tozsin et al. (2024)	Systematic Review	AMSTAR 2	High	Variable study quality and limited longitudinal evidence in medical education.
29	Hallquist et al. (2025)	Systematic Review	AMSTAR 2	Moderate	Heterogeneous assessment approaches and limited evidence of transfer to clinical practice.
30	Shishehgar et al. (2025)	Systematic Review	AMSTAR 2	High	Reliance on self-reported perceptions and substantial contextual heterogeneity.
31	Luo et al. (2025)	Systematic Review and Meta-Analysis	AMSTAR 2	Moderate	Readiness outcomes were mainly self-reported and measured using heterogeneous instruments.
32	Garzon et al. (2025)	Systematic Review	AMSTAR 2	High	Broad educational scope and variability in methodological quality across studies.
33	Arar et al. (2025)	Systematic Review	AMSTAR 2	Moderate	Limited empirical validation of leadership and management outcomes.
34	Rangel-de Lazaro & Duart (2023)	Systematic Review	AMSTAR 2	High	Heterogeneity in extended-reality technologies and limited long-term outcome evidence.
35	Gado et al. (2022)	Cross-Sectional Survey Study	JBI Analytical Cross-Sectional Checklist	Moderate	Single-country student sample and self-reported acceptance measures limited external validity.
36	Parmigiani et al. (2022)	Systematic Review	AMSTAR 2	Moderate	Heterogeneity in violence-risk samples, predictors, algorithms, and validation procedures limited clinical generalizability.
37	Rogan et al. (2024)	Systematic Review with Meta-Synthesis	AMSTAR 2	High	Heterogeneity in clinician populations, passive sensing technologies, and implementation contexts limited direct comparisons and generalizability.
38	Gooding & Kariotis (2021)	Scoping Review	JBI Checklist for Systematic Reviews and Research Syntheses	High	Legal and ethical evidence was heterogeneous and mainly non-empirical.
39	Ienca & Ignatiadis (2020)	Narrative Review	SANRA	High	Conceptual ethical analysis with limited prospective evaluation of proposed safeguards.
40	Walsh et al. (2020)	Perspective / Recommendations Article	JBI Checklist for Text and Opinion Papers	Moderate	Recommendations on bias and stigma were conceptually grounded but not prospectively tested.
41	Fiske et al. (2020)	Commentary / Ethical Analysis	JBI Checklist for Text and Opinion Papers	Moderate	Proposed embedded-ethics approach lacked empirical implementation evaluation.
42	Jacobson et al. (2020)	Ethical and Policy Analysis	JBI Checklist for Text and Opinion Papers	Moderate	Ethical and policy recommendations were not tested in comparative or prospective studies.
43	Reddy et al. (2020)	Governance Framework Article	JBI Checklist for Text and Opinion Papers	Moderate	Governance framework lacked prospective organizational or real-world validation.
44	Gerke et al. (2020)	Narrative Review	SANRA	High	Legal analysis was comprehensive but primarily conceptual rather than empirical.
45	Char et al. (2020)	Qualitative Ethics Study	JBI Checklist for Qualitative Research	Moderate	Stakeholder-derived ethical themes require broader validation across settings and populations.
46	Mörch et al. (2020)	Framework Development and Delphi Validation Study	MMAT	Moderate	Expert Delphi validation was limited by panel size and absence of real-world implementation testing.
47	Straw & Callison-Burch (2020)	Empirical Algorithmic-Bias Study	JBI Analytical Cross-Sectional Checklist	Moderate	Bias was demonstrated in selected language models and datasets; broader clinical generalizability remains uncertain.
48	Crossnohere et al. (2022)	Literature Review	SANRA	Moderate	Heterogeneous value frameworks and limited evidence of practical implementation.
49	Prakash et al. (2022)	Scoping Review	JBI Checklist for Systematic Reviews and Research Syntheses	Moderate	Broad ethical scope and limited assessment of the effectiveness of governance responses.
50	Rubeis (2022)	Narrative Review	SANRA	High	Primarily philosophical analysis with limited empirical evaluation.
51	Salah et al. (2024)	Narrative Review / Perspective	SANRA	High	Rapidly evolving evidence and limited prospective evaluation of GPT-related risks.

No.	First Author (Year)	Verified Study Design	Appraisal Tool	Overall Appraisal	Main Methodological Limitation / Action
52	Tavory (2024)	Ethics and Regulatory Perspective	JBI Checklist for Text and Opinion Papers	Moderate	Normative regulatory argument was not empirically validated in mental-health services.
53	Saeidnia et al. (2024)	Narrative Ethical Review	SANRA	Moderate	Recommendations were not supported by extensive prospective implementation evidence.
54	Ortega-Bolaños et al. (2024)	Systematic Review	AMSTAR 2	High	Considerable heterogeneity among ethical-assessment tools and validation approaches.
55	Batool et al. (2025)	Systematic Review	AMSTAR 2	High	Governance mechanisms varied considerably across sectors and jurisdictions.
56	Robles & Mallinson (2025)	Systematic Review	AMSTAR 2	High	Unified framework requires empirical validation across different governance contexts.
57	Ismail & Ahmad (2025)	Systematic Review	AMSTAR 2	Moderate	Heterogeneous frameworks and limited comparative assessment of implementation effectiveness.
58	Blease & Rodman (2025)	Ethical Evaluation / Narrative Review	SANRA	High	Ethical conclusions were mainly conceptual and based on a rapidly changing evidence base.
59	Abusamra et al. (2025)	Systematic Review	AMSTAR 2	Moderate	Substantial heterogeneity across included studies and limited evidence for real-world implementation.
60	Vilaza & McCashin (2021)	Ethical Framework Analysis	JBI Checklist for Text and Opinion Papers	Moderate	Ethical framework application did not directly evaluate clinical effectiveness or safety outcomes.
61	Murphy et al. (2021)	Scoping Review	JBI Checklist for Systematic Reviews and Research Syntheses	Moderate	Broad thematic mapping with limited appraisal of effectiveness or causal evidence.
62	Tangsrivimol et al. (2025)	Narrative Review	SANRA	Moderate	Limited empirical validation and uncertainty regarding the safe implementation of ChatGPT in healthcare settings.
63	Chandler (2020)	Narrative Review	SANRA	Moderate	Narrative discussion with limited empirical evidence supporting real-world implementation and effectiveness.
64	Boucher et al. (2021)	Narrative Review	SANRA	High	Heterogeneity in chatbot interventions and limited evidence for long-term clinical outcomes.
65	Shatte et al. (2019)	Systematic Scoping Review	JBI Checklist for Systematic Reviews and Research Syntheses	High	Considerable heterogeneity in machine-learning methods, datasets, and reported outcomes.
66	Mohr et al. (2021)	Secondary Analysis of Three Clinical Trials	JBI Cohort Checklist	Moderate	Secondary pooled analysis depended on the design and reporting quality of the underlying trials.
67	Singhal et al. (2023)	Benchmark and Human-Evaluation Study	JBI Analytical Cross-Sectional Checklist	Moderate	Benchmark and expert evaluation did not establish real-world mental-health effectiveness or safety.
68	Chivilgina et al. (2020)	Systematic Review	AMSTAR 2	Moderate	Heterogeneous mobile-health interventions and limited evidence of long-term clinical effectiveness.
69	Chivilgina et al. (2021)	Narrative Review	SANRA	Moderate	Descriptive synthesis with limited systematic appraisal of digital interventions.
70	Graham et al. (2025)	Commentary / Practice Guidance	JBI Checklist for Text and Opinion Papers	Moderate	Advocacy-oriented conclusions were not evaluated through comparative empirical analysis.
71	Jermutus et al. (2022)	Narrative Review	SANRA	Moderate	Broad descriptive synthesis with limited prospective validation and heterogeneous evidence across studies.
72	Zhou et al. (2022)	Literature Review	SANRA	Moderate	Broad descriptive coverage with limited evaluation of clinical validation and study quality.
73	Torous et al. (2021)	State-of-the-Art Review	SANRA	High	Broad digital-psychiatry synthesis with limited quantitative comparison of implementation outcomes.
74	Coghlan et al. (2023)	Critical Ethical Review	SANRA	High	Ethical analysis was comprehensive but lacked prospective testing of proposed safeguards.
75	Demszky et al. (2023)	Interdisciplinary Review with Demonstration Studies	SANRA	High	Rapidly evolving evidence base and limited validation across diverse psychological contexts.
76	Denecke et al. (2021)	System Development and Usability Study	JBI Checklist for Analytical Cross-Sectional Studies	Moderate	Small or selected usability sample and absence of long-term clinical effectiveness evaluation.
77	Garcia-Martínez et al. (2023)	Narrative Review	SANRA	Moderate	Narrative synthesis highlighting emerging opportunities, with limited evidence from prospective clinical validation.
78	Garg et al. (2023)	Systematic Review	AMSTAR 2	Moderate	Highly heterogeneous applications and limited high-quality clinical evidence for ChatGPT.

No.	First Author (Year)	Verified Study Design	Appraisal Tool	Overall Appraisal	Main Methodological Limitation / Action
79	Garriga et al. (2022)	Prediction-Model Development and Prospective Clinical Evaluation Study	PROBAST	Moderate	Prospective evaluation was promising, but transportability, workflow effects, and broader external validation remain limited.
80	Guo et al. (2024)	Systematic Review	AMSTAR 2	Moderate	Included LLM studies were early-stage, heterogeneous, and used nonstandardized validation methods.
81	Lee et al. (2021)	Narrative Review	SANRA	High	Strong conceptual synthesis but limited prospective evidence of routine clinical implementation.
82	Lomis et al. (2021)	Educational Perspective / Guidance Article	JBI Checklist for Text and Opinion Papers	Moderate	Curricular recommendations were not tested against direct learner or competency outcomes.
83	Malgaroli et al. (2023)	Systematic Review and Research Framework	AMSTAR 2	Moderate	NLP intervention studies were methodologically heterogeneous and showed limited replication and external validation.
84	Meskó & Topol (2023)	Regulatory Perspective	JBI Checklist for Text and Opinion Papers	Moderate	Regulatory proposals were conceptual and lacked empirical implementation evaluation.
85	Obradovich et al. (2024)	Narrative Review	SANRA	High	Limited prospective psychiatric evaluation and uncertainty regarding safety in high-risk use.
86	Omiyefa (2025)	Narrative Review	SANRA	Moderate	Review conclusions were based primarily on conceptual evidence with limited empirical validation.
87	Scholich et al. (2025)	Comparative Study	JBI Checklist for Quasi-Experimental Studies	Moderate	Comparative evaluation was limited by sample characteristics and short-term assessment of chatbot performance.
88	Welch et al. (2022)	Scoping Review	JBI Checklist for Systematic Reviews and Research Syntheses	Moderate	Evidence was heterogeneous, and long-term effectiveness of mobile AI interventions remains insufficiently established.

Revised methodological quality assessment text for Section

Methodological quality was independently assessed by two reviewers using validated appraisal tools appropriate to each verified study design. Systematic reviews and meta-analyses were assessed using AMSTAR 2, scoping and systematic-scoping reviews using the relevant JBI critical appraisal criteria, and narrative reviews using SANRA. Prediction-model development and validation studies were assessed using PROBAST. Observational and cross-sectional studies were evaluated using the appropriate JBI checklist, qualitative studies using the JBI Checklist for Qualitative Research, and text, opinion, commentary, perspective, and governance-framework articles using the JBI Checklist for Text and Opinion Papers. Mixed-design and Delphi-based studies were assessed using MMAT where appropriate. Each study was evaluated independently by two reviewers; disagreements were resolved through discussion and, when necessary, consultation with a third reviewer. Quality-assessment findings informed interpretation of the evidence but were not used as exclusion criteria.

Interpretive note below Table S2

The verified appraisal framework demonstrated substantial methodological diversity across the included evidence. Review-level studies were evaluated using AMSTAR 2, JBI, or SANRA according to review design, whereas prediction models, observational studies, qualitative studies, and conceptual or governance articles were evaluated using the appraisal tools most appropriate to their verified study designs. Common methodological limitations included heterogeneity, limited external validation, small or selected samples, reliance on retrospective evidence, and limited real-world implementation.

Abbreviations: AMSTAR 2, A MeaSurement Tool to Assess Systematic Reviews 2; JBI, Joanna Briggs Institute Critical Appraisal Checklist(s); MMAT, Mixed Methods Appraisal Tool; PROBAST, Prediction model Risk Of Bias ASsessment Tool; SANRA, Scale for the Assessment of Narrative Review Articles.
